# Prevalence of Parental Alcohol Problems among a General Population Sample of 28,047 Norwegian Adults: Evidence for a Socioeconomic Gradient

**DOI:** 10.3390/ijerph18105412

**Published:** 2021-05-19

**Authors:** Siri H. Haugland, Tobias H. Elgán

**Affiliations:** 1 Department of Psychosocial Health, University of Agder, 4876 Grimstad, Norway; 2 STAD, Centre for Psychiatry Research, Department of Clinical Neuroscience, Karolinska Institutet & Stockholm Health Care Services, Region Stockholm, 113 64 Stockholm, Sweden; tobias.elgan@ki.se

**Keywords:** parental alcohol use, social gradient, Children of Alcoholics Screening Test (CAST-6), prevalence, adult survivors of adverse life events

## Abstract

The aim of the study presented here was to estimate the prevalence of parental alcohol problems during childhood in a general population of Norwegian adults, and to investigate associations between parental alcohol problems during childhood and lower socioeconomic status in adulthood. This cross-sectional study recruited 28,047 adults (≥18 years) to an online health survey (Norwegian Counties Public Health Surveys). We evaluated demographic and socioeconomic measures and responses to a shortened version of the Children of Alcoholics Screening Test (CAST-6) scale to assess whether respondents perceived parental alcohol consumption during childhood as problematic. Respondents reported parental alcohol problems at a rate of 15.6%, but the experience was more prevalent among adults with a low education (20.0%), compared to those with intermediate (16.4%) or high educations (13.8%, *χ^2^*(2) = 87.486, *p* < 0.001), and it was more common among respondents with low economic capabilities (21.1%) compared to those with middle/high capabilities (14.2%, *χ^2^*(1) = 162.089, *p* < 0.001). Parental alcohol problems were most prevalent among respondents that received welfare benefits (24.5%). Multivariable logistic regression analyses revealed associations between parental alcohol problems and low socioeconomic status in adulthood; odds ratios (95% confidence intervals) ranged from 1.33 (1.25–1.42) to 1.89 (1.72–2.06). From a public health perspective, children who grow up with parental alcohol problems should be reached through both universal and selective interventions.

## 1. Introduction

Children who grow up in families where the parents have alcohol problems are at increased risk of several negative consequences, including poor school performance, poor mental health, and early onset alcohol use [1,2,3,4,5,6]. Parentification may also occur, where children assume adult roles even though they are not developmentally or emotionally ready [7]. The consequences are often long-term [8], and they augment the likelihood of other disorders, for instance, mental disorders such as major depression [9]. Furthermore, studies have demonstrated that when parents have alcohol problems, their offspring are at increased risk of alcohol-related hospitalization and mortality, including suicide [10,11].

Currently, international studies have estimated that the prevalence of children with parents who have alcohol problems is 4–29% [12,13,14,15,16,17]. The primary reason for this broad range is that parental alcohol problems are defined and assessed differently in different studies. For instance, some studies examined hazardous drinking among parents and others examined parental alcohol use disorder. Furthermore, some studies were based on self-reports from either the children or parents, and others were based on surveys, psychiatric interviews, or registry data. Drinking patterns vary across countries, and there may also be differences in how alcohol problems are defined.

In Nordic countries, only a handful of scientifically determined estimates are available, and the estimated prevalence varies. A web-based survey distributed to Swedish youth, 16–19 years old, concluded that 20.1% of the sample had at least one parent with an alcohol problem [13]. In that study, perceived alcohol problems were assessed with the short version of the Children of Alcoholics Screening Test (CAST-6) [18]. Another survey, which was distributed to a nationally representative sample of Swedish adults, 17–84 years old, assessed alcohol problems with the Mini International Psychiatric Interview, derived from the Diagnostic and Statistical Manual of Mental Disorders, fourth edition. They concluded that 3.7% of children had at least one parent with a current alcohol use disorder [12]. Another study, based on Danish registry data, concluded that 4.5% of children had parents that had been hospitalized due to an alcohol-related illness [17]. A recent Danish study, based on 75,853 high-school and vocational school students, reported that 7.3% of the surveyed students perceived that they had at least one parent with alcohol problems [19]. A Norwegian study, based on reports from parents of teenagers, found that 15.6% of fathers and 4.7% of mothers were defined as individuals that misused alcohol [20]. However, these figures may not be generalizable to parents with younger children [21]. The scarcity of data on the prevalence of children who have parents with alcohol problems in Norway calls for further studies.

Early adversity may have a negative impact on many aspects of life, including socioeconomic indicators, such as education, employment, and income [22]. However, to the best of our knowledge, no studies have explicitly investigated whether there exists a social gradient connected to parental alcohol problems in non-clinical populations. Moreover, although it is important to understand how widespread parental alcohol problems are, it would be valuable to have estimates based on the perceptions of the children or adult children. Therefore, this study aimed to estimate the prevalence of parental alcohol problems during childhood in a general population of Norwegian adults, and to investigate associations between parental alcohol problems during childhood and lower socioeconomic status in adulthood.

## 2. Materials and Methods

This cross-sectional study included a random sample of 75,191 individuals, aged 18 years or older, that resided in the region of Agder (30 municipalities in southern Norway). The sample was drawn from the Norwegian Population Registry, and e-mails or telephone numbers were obtained from the contact registry of the Agency for Public Management and eGovernment (Difi). Individuals who had declined to participate in surveys, individuals registered as deceased, those with unverified contact information, and those with an address outside the region were removed. Thus, in 2019, 61,611 inhabitants were invited to participate in the Norwegian Counties Public Health Survey. The respondents participated by completing a questionnaire online. The questionnaire included questions related to health, well-being, childhood, living conditions, local environments, accidents, and injuries. Participants gave online consent to participate when they answered the survey questions, and provided their age and sex to confirm their identity. Of the 61,611 invited individuals, 28,047 completed the questionnaire; the response rate was 45.5%.

### 2.1. Ethics

Informed consent was obtained from all subjects involved in the study. All personal identification variables were removed before the researchers obtained the dataset. Data were handled in compliance with applicable personal data protection regulations. The Norwegian Institute of Public Health (Oslo) is responsible for the health survey. The survey was approved by the Norwegian Data Inspectorate, and it adhered to the regulations of the Personal Health Data Filing System Act. In addition, a Data Protection Impact Assessment was performed by the Norwegian Institute of Public Health. Ethical approval for the current study was obtained from The National Committees for Research Ethics in Norway (REK) (file number 162353), and from the Faculty Ethics Committee at the University of Agder.

### 2.2. Measures

The questions, response categories, and definitions used in the survey are shown in Table 1.

The six-item CAST-6 instrument (Table 1) was used to estimate perceived parental alcohol problems [18]. Respondents could answer yes = 1 or no = 0 to each question, and the total score ranged from 0 to 6. The CAST-6 demonstrated high internal consistency (α = 0.86–0.92) and concurrent validity (r = 0.93), compared to the original 30-item CAST for adults [18,23,24]. Moreover, it showed good (r = 0.78) to excellent (r = 0.94, ICC = 0.93) test-retest reliability for both adults and adolescents [23,24,25]. In the present study, the scale showed excellent reliability (α = 0.91).

Two alternative cut-off scores are commonly used with the CAST-6. One cut-off score is more inclusive (2 points) and the other is more conservative (3 points) [18,24,26,27]. The more conservative cut-off score was used in the current study. Data on socioeconomic factors were collected with questions related to education, economic capability, employment status, and whether respondents received welfare benefits (disability pension/work assessment allowance/social assistance benefits). Participants’ age and sex were provided through the national population registry. In addition, participants were asked about their marital status.

### 2.3. Statistical Analysis

Data were analysed with SPSS version 25 (SPSS Inc., Chicago, IL, USA). Descriptive statistics for the overall sample were estimated for key demographic and socioeconomic variables. Pearson’s χ^2^ analyses were performed to evaluate associations between the overall distribution of parental alcohol problems and the demographic and socioeconomic variables. Multivariable logistic regression was performed to investigate the association between parental alcohol problems and measures of low socioeconomic status, adjusted for age and sex. Results are expressed as odds ratios (OR) with 95% confidence intervals (95% CI). A *p*-value < 0.05 was considered statistically significant.

## 3. Results

Descriptive characteristics of the sample are provided in Table 2.

Table 3 shows that, overall, 15.6% of the respondents had experienced problematic alcohol use among their parents during childhood. This experience was significantly more prevalent among females (17.5%) than among males (13.4%; *p* < 0.001). The proportion of individuals who reported experiences of problematic parental alcohol use varied among different age groups. The lowest prevalence was observed for respondents aged 67 years or older. Moreover, this experience was less common among respondents that were married or had a registered partner, compared to those with another relationship status. We also observed a consistent social gradient in associations between parental alcohol problems and various socioeconomic variables. Parental alcohol problems were more prevalent among those with a lower education level, compared to those with intermediate or high education levels; among those with low economic capability, compared to those with middle/high economic capability; among those on sick leave, compared to those not on sick leave; and among those who received welfare benefits, compared to those who did not receive welfare benefits.

**Figure 1:**
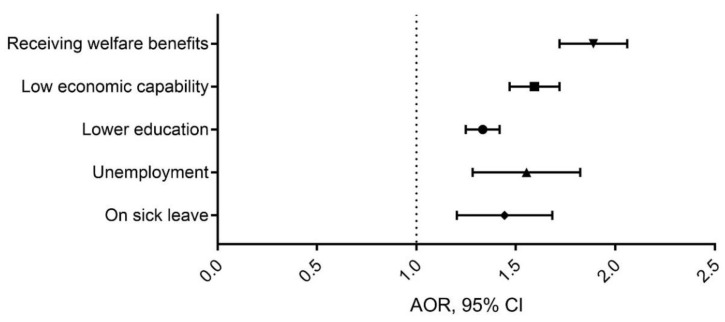
Associations between parental alcohol problems and measures of low socioeconomic status, based on the Agder County Public Health Study (Norway 2019). Data are results from a multivariable logistic regression analysis, adjusted for sex and age. Values represent adjusted odds ratios (AOR) and 95% confidence intervals (95% CI).

Results from the multivariable logistic regression are displayed in Figure 1. Findings revealed consistent associations between parental alcohol problems and all measures of low socioeconomic status. The strongest association was found between parental alcohol problems and the need for welfare benefits (OR: 1.89, 95% CI: 1.72–2.06; *p* < 0.001). Other forms of marginalization within the work force, such as being on sick leave or being unemployed, were also associated with parental alcohol problems (OR: 1.42, 95% CI: 1.21–1.69; *p* < 0.001; and OR: 1.54, 95% CI: 1.47–1.72; *p* < 0.001, respectively). The experience of parental alcohol problems was also significantly associated with no college/university education (OR: 1.33, 95% CI: 1.25–1.42, *p* < 0.001).

## 4. Discussion

We found that, among an adult Norwegian sample randomly drawn from the general population, 15.6% had experienced problematic parental alcohol use during childhood. To the best of our knowledge, no previous studies have estimated the prevalence of parental alcohol problems in the Nordic context based on a broad age range of adult offspring from the general population. A previous Norwegian study analysed self-reported problems with alcohol use among parents of teenagers. They found that 15.6% of the fathers had alcohol problems (scores ≥2 with the CAGE screening instrument), but the proportion of mothers in this category was significantly lower (4.7%). In our study, we did not group individuals based on parental sex; thus, our finding that 15.6% of parents had problematic drinking behaviours included fathers, mothers, or both.

Other international estimates of the prevalence of parental alcohol problems have varied greatly (4–29%) [12,13,14,15,16,17]. This variation might partly be explained by differences in the samples and measures used in different studies. A Swedish study included adolescents aged 16–19 years, and also used the CAST-6. They found that the prevalence of respondents that reported perceived parental alcohol problems was 20.1% [13], which was somewhat higher than our estimate. This difference might be explained by the difference in respondents’ age between studies.

Our results indicated that the oldest age group (aged 67+ years) was least likely to report parental alcohol problems. This result could be explained by several factors. First, the questions were retrospective in nature, and recall bias could be a prominent issue [28]. Second, it has been shown that adverse childhood experiences, such as parental alcohol problems, were associated with impaired health [29] and elevated mortality [10]; therefore, the oldest respondents who experienced problematic parental alcohol problems could have been underrepresented. Third, alcohol consumption among Norwegian adults increased after the second world war [30]; thus, it is plausible that the prevalence of parental alcohol problems was, in fact, relatively low during the era that the oldest participants grew up.

We also found that parental alcohol problems were reported slightly more frequently by females than by males. Although this result was puzzling, other studies have shown similar findings [24,25]. Havey and Dodd [25] have suggested that females, compared to males, may be more sensitised toward substance use and related issues within the family, and that they also may be more prone to express concern about a family situation in a self-reported questionnaire.

Overall, our findings showed that perceived parental alcohol problems were most prevalent among socioeconomically disadvantaged groups (i.e., individuals with low education levels, low economic capability, or a need for welfare benefits). The largest proportion of respondents that experienced parental alcohol problems comprised those who received a disability pension, work assessment allowance, or social assistance benefits (welfare benefits). In this group of respondents, 25% experienced parental alcohol problems during childhood. This finding remained significant after adjusting for age and sex in the multivariable analyses. Although alcohol consumption in Norway was found to be highest among adults with a high education level [31], we found that the respondents’ childhood experiences of problematic parental alcohol use were inversely associated with the respondents’ education level. Other studies have also found socioeconomic inequalities in the distribution of individuals that experienced alcohol-related harm [32]. Although we lack studies that have specifically addressed socioeconomic differences in the distribution of individuals with parental alcohol problems, other studies have shown that adverse life experiences are socially patterned in childhood [33]. Therefore, the social gradient that we observed among our adult respondents could be related to the socioeconomic disadvantage present in childhood. However, adverse childhood experiences can also reduce educational attainment; indeed, Houtepen et al. [34] found that this relationship remained significant after controlling for family socioeconomic variables. Possible explanations of these relationships are likely complex. Exposure to chronic stress may induce changes in the developing brain and impact a range of important functions that interfere with learning and other skills needed to succeed in education or the workplace [35]. Childhood adversities such as parental alcohol problems could also increase health risk behaviours, physical and mental health problems, and developmental disruptions [36] which may also contribute to economic marginalisation.

### 4.1. Study Strengths and Limitations

This study has expanded existing knowledge by contributing estimates of perceived parental alcohol problems, based on reports from a large adult sample of 28,047 individuals drawn randomly from the general population. Our outcome was based on the CAST-6, which is a validated instrument [24]. Item four of the CAST-6 presumes the presence of two parents, which could influence the score for respondents who grew up in single-parent families. Sensitivity analyses excluding this item did not alter the findings significantly. Our findings shed light on the socioeconomic patterns associated with the prevalence of parental alcohol problems, which were rarely studied in previous research.

This study was limited by its retrospective design. Moreover, responses could be prone to recall bias and the risk of measurement error [28]. The validity of retrospective assessments of childhood experiences has been debated; however, a comparison between retrospective reports and prospective results did not reveal a bias in the retrospective assessment of difficult childhood experiences [37]. Additionally, cautiousness regarding the generalizability of the findings is necessary due to possible non-response bias. Finally, this study was based on cross-sectional data; therefore, the results should be interpreted with caution when considering causality.

### 4.2. Implication for Practice

The CAST-6 was not designed to identify diagnostic criteria; instead, it identifies individual perceptions of problematic parental alcohol use. Previous studies that investigated adverse outcomes related to parental non-dependent alcohol use had mainly focused on offspring substance use [38]. However, several studies have identified other negative outcomes related to parental non-dependent drinking patterns [39,40,41]. These dysfunctional patterns often continue into the next generation. To break the patterns, early support interventions should be available. However, support might be available to varying degrees. For instance, in Sweden, the vast majority of municipal social services provide support to children growing up with parental substance use problems, most often in the form of individual counselling or support groups but, at the same time, support only reaches a small proportion of the targeted children [42]. Several organizations identify children in need and offer support, including the adult substance-use treatment services, psychiatric care, and social services. However, studies have shown that, in most cases, those organizations did not determine whether the clients had children [43]. The situation in Norway appears to be similar: only about one fifth of the professionals working in substance use treatment facilities offered support to their clients’ children, and about half of the professionals never assessed whether the clients had children [44]. One obvious arena to identify children in need of support is the school setting. Since these children often are neglected, schools could work with policy documents and action plans and inform and train their staff about this vulnerable group. Previous research has shown that policy documents increase the likelihood of school staff to receive training in this issue, which in turn increases the likelihood of identifying these vulnerable children in the school setting [45]. Digital interventions represent a promising approach for increasing the availability of support. However, currently, only a small number of digital interventions are currently being tested that target this group of individuals [46,47,48,49]. For instance, in Sweden an online chat group program has been developed [47], based on a Dutch program [48]. The program consists of eight weekly sessions, each 60–90 min long, focusing on themes such as ‘your role in the family’, ‘social networks’, and ‘substance use, tolerance, and heredity’. Each session is moderated by a trained counsellor. The program is currently being evaluated but has the potential to reach a large number of adolescents and young adults.

## 5. Conclusions

This study showed that one in six adults reported problematic parental alcohol use and, among disadvantaged sub-groups, this prevalence increased to one in four. It is imperative to make both universal and selective prevention interventions available at an earlier age if we expect to break family patterns of problematic alcohol consumption. In addition, we need better methods for early detection, for instance by identifying burdened children when parents are in contact with general or more specialized health care [43,50]. Furthermore, we should ensure proper support and follow-up for these children and their families.

## Figures and Tables

**Table 1:** Norwegian Counties Public Health Survey, conducted in Agder, 2019.

Variable	Questions	Response Options	Category Definitions
**Problematic parental alcohol use**	**CAST-6 items**	Yes/no	A sum score ≥3 on the CAST-6 was defined as parental alcohol problems vs. score <3; not a problem
	Have you ever thought that one of your parents had a drinking problem?		
	2. Did you ever encourage one of your parents to quit drinking?		
	3. Did you ever argue or fight with a parent when he or she was drinking?		
	4. Have you ever heard your parents fight when one of them was drunk?		
	5. Did you ever feel like hiding or emptying a parent’s bottle of liquor?		
	6. Did you ever wish that a parent would stop drinking?		
**Sex**	Sex	Retrieved from registries	Male/female
**Age**	Age	Retrieved from registries	Age groups: 18–24, 25–44, 45–66, 67+ yearsIn multivariable analyses, age was employed as a continuous variable
**Marital status**	What is your marital status?	Married/registered partner Cohabitee Have a girlfriend/boyfriend (but do not live together Single	Partner vs. single
**Education**	What is your highest completed level of education?	Lower secondary/secondary modern/folk high school ≤ 10 y Vocational training/middle school /upper secondary/junior college University/college <4 years University/college ≥4 years	1 = low education 2 = intermediate education 3 and 4 = higher education
**Financial capabilities**	For one-person households, consider your total income. If you live with others, consider the total income of everyone in the household. How easy or difficult is it for you to make ends meet day to day with this income?	Very difficult Difficult Relatively difficult Relatively easy Easy Very easy Do not know	1–3 = low economic capability vs. 4–7 = middle/high economic capability
**Employment status**	What is your current status concerning employment etc.?(Select as many as applicable.)	Full-time Part-time Self-employed, On sick leave Unemployed Receiving disability pension/work assessment allowance Receiving social assistance benefits In retirement/early retirement Pupil/student Undertaking national/alternative civilian service Homemaker	1 = ≥ 32 h/week vs. not2 = < 32 h/week vs. not3 = Self-employed vs. not4 = On sick-leave vs. not6 and 7 = Receiving welfare benefits vs. not

**Table 2:** Demographic and socioeconomic statistics from the Norwegian Counties Public Health Survey, in Agder, 2019.

Characteristics	n	%
**Sex**		
Female	14,925	53.2
Male	13,122	46.8
**Age group, y**		
18–24	3169	11.3
25–44	9180	32.7
45–66	12,026	42.9
67+	3672	13.1
**Education level**		
Low	3333	11.9
Intermediate	11,088	39.7
High	13,502	48.4
Employment status *		
Difficult financial situation	5547	20.8
Easy financial situation	21,140	79.2
Employed full-time	14,278	50.9
Employed part-time	3840	13.7
Self-employed	1470	5.2
Unemployed	815	2.9
On sick leave	849	2.9
On welfare benefits	3208	11.4

* Multiple responses could be selected; education level and employment status are defined in Table 1.

**Table 3:** Prevalence of perceived parental alcohol problems, stratified by demographic and socioeconomic background variables from the Norwegian Counties Public Health Survey in Agder, 2019.

Characteristics	Parental Alcohol Problems (CAST-6 Sum Score ≥ 3)			
	N (%)	χ^2 (*a*)^	*df*	*p*
Female	2597 (17.5)	88.13	1	<0.001
Male	1759 (13.4)			
Age 18–24 y	406 (12.9)	123.19	3	<0.001
Age 25–44 y	1587 (17.4)			
Age 45–66 y	1983 (16.6)			
Age 67+ y	370 (10.2)			
Married/registered partner	2107 (14.1)	66.84	3	<0.001
Cohabitee	871 (18.8)			
Have a girlfriend/boyfriend	371 (16.7)			
Single	989 (16.4)			
Low education	660 (20.0)	87.49	2	<0.001
Intermediate education	1814 (16.4)			
High education	1856 (13.8)			
Low economic capability	1176 (21.1)	162.09	1	<0.001
High economic capability	2999 (14.2)			
Employed full-time	2107 (15.4)	1.05	1	0.305
vs. not employed full-time	2239 (15.8)			
Employed part-time	640 (16.7)	4.60	1	0.032
vs. not employed part-time	3706 (15.4)			
Self-employed	194 (13.2)	6.62	1	0.010
vs. not self-employed	4152 (15.7)			
Unemployed	176 (21.6)	23.30	1	<0.001
vs. not unemployed	4170 (15.4)			
On sick leave	181 (21.4)	22.63	1	<0.001
vs. not on sick leave	4165 (15.4)			
Welfare benefits	782 (24.5)	218.19	1	<0.001
vs. no welfare benefits	3564 (14.4)			
Total	4346 (15.6)			

**^(*a*)^** Categories (defined in Table 1) were compared with Pearson’s χ^2^ test; education levels, economic capabilities, and employment statuses are defined in Table 1.

## Data Availability

Restrictions apply to the availability of these data. Data was obtained from Norwegian Institute of Public Health (NIPH) and are available at https://helsedata.no/en (accessed on 27 April 2021) with the permission of NIPH.
